# Skeleton-Based Abnormal Gait Detection

**DOI:** 10.3390/s16111792

**Published:** 2016-10-26

**Authors:** Trong-Nguyen Nguyen, Huu-Hung Huynh, Jean Meunier

**Affiliations:** 1 DIRO, University of Montreal, Montreal, QC H3T 1J4, Canada; meunier@iro.umontreal.ca; 2 The University of Danang - University of Science and Technology, Danang 556361, Vietnam; hhhung@dut.udn.vn

**Keywords:** human gait, gait analysis, gait cycle, hidden Markov model, Kinect

## Abstract

Human gait analysis plays an important role in musculoskeletal disorder diagnosis. Detecting anomalies in human walking, such as shuffling gait, stiff leg or unsteady gait, can be difficult if the prior knowledge of such a gait pattern is not available. We propose an approach for detecting abnormal human gait based on a normal gait model. Instead of employing the color image, silhouette, or spatio-temporal volume, our model is created based on human joint positions (skeleton) in time series. We decompose each sequence of normal gait images into gait cycles. Each human instant posture is represented by a feature vector which describes relationships between pairs of bone joints located in the lower body. Such vectors are then converted into codewords using a clustering technique. The normal human gait model is created based on multiple sequences of codewords corresponding to different gait cycles. In the detection stage, a gait cycle with normality likelihood below a threshold, which is determined automatically in the training step, is assumed as an anomaly. The experimental results on both marker-based mocap data and Kinect skeleton show that our method is very promising in distinguishing normal and abnormal gaits with an overall accuracy of 90.12%.

## 1. Introduction

Musculoskeletal disorders are one of the leading causes of physician visits and have a major impact on society in terms of long-term disability and economics. As the population is aging in several countries, these problems will rapidly increase in the future. This paper focuses on the problem of detecting abnormal human gait in walking mode in clinical medical practice. Because there is no exact definition of an abnormal gait, our work simply considers it as a significant deviation from the range of normal gait learned with a model. A gait cycle is thus considered abnormal if its normality likelihood (obtained with our trained model) is less than a threshold. Consequently, many solutions were proposed to estimate musculoskeletal conditions with the support of technologies. In particular, gait analysis has shown a lot of evidence demonstrating its potential for clinical medical practice. For that matter, many recent studies employ wearable infrared and/or acceleration sensors in order to measure physical parameters of human gait such as stride length or velocity. For instance, Greene et al. [1] were able to distinguish patients with a history of falls based on a variety of parameters that are measured using body-worn kinematic sensors. Another example is the work of Chen et al. [2] where human abnormal gait was modeled using a hidden Markov model (HMM) combined with information from sensors attached inside the shoes.

Although these approaches provide good accuracies since average success rate was 76.8% for [1] and 88.6% for [2], the device price could become an issue of concern. In addition, an important disadvantage is that it is inconvenient for the patients to wear these devices on their body. In order to avoid these limitations, vision-based techniques were proposed to perform gait analysis using data captured via calibrated RGB (Red, Green, Blue) cameras or devices with depth sensors (RGB-D cameras) such as the Microsoft Kinect (Redmond, WA, USA) [3].

Some studies deal with gait analysis using only one camera. For instance, the authors in [4] propose a clinical system to measure 2D angles between major body segments with an ellipse-based hierarchical tree structure. However, the system is limited to fronto-parallel (side view) gait only and does not provide fully 3D measurements. Therefore, we decided to use the Microsoft Kinect, a depth camera with built-in functions providing in-scene human skeletons. Although this camera works best with frontal view, the obtained skeleton, especially lower body joints, is acceptable at a reasonable (up to $\pm 60^{\circ }$ [5]) angle from a frontal view for gait analysis. Other researchers have relied on optical flow and variants. In [6], the researchers employed optical flow, normalized flow histograms, principal component analysis (PCA) and variants of the Hausdorff distance together with the nearest neighbor technique for classification. One disadvantage of this approach is that it is time-consuming due to high computational cost. Furthermore, its generalization level cannot be guaranteed because the algorithm was tested with two datasets consisting of only one subject each. Finally, 3D information is not available. In [7], the silhouette shape is encoded using lattices. They are incorporated into a temporal context by basically concatenating feature vectors obtained for each frame. Each movement pattern is then classified using a support vector machine (SVM) algorithm. The accuracy in determining unusual motion was around 70%–80% but could be impaired significantly by outlier frames resulting from ambiguities due to the 2D projection of the body and loss of 3D information.

Other approaches use 3D information for better gait analysis. For instance, Stone and Skubic [8] extracted gait cues based on the human silhouettes captured via a pair of calibrated cameras or a Kinect camera. In a sequence of frames corresponding to human walking, the positions of footfalls are localized based on such 3D reconstruction (pair of cameras) or depth information (Kinect). Temporal and spatial gait parameters are then estimated. Our approach also employs 3D information, but this is extracted from the skeletal model provided by the Kinect. The skeletal model was not used in [8] because its estimation is possible only in a limited range that was insufficient for in-home monitoring.

Finally, other methods take full advantage of the 3D skeleton provided by the Kinect. Paiement et al. [9,10] proposed an online system for estimating the movement quality from Kinect skeleton. They built a statistical model for representing the normal movement which was captured from healthy subjects. The assessment stage was then performed based on each frame as well as each sequence following Markov assumptions. The study [10] evaluated four models including three discrete-state HMMs and a continuous-state HMM. Our model is closer to the first one (named $\lambda_{a}$ in [10]), the main difference being the discrete (vs. continuous) nature of observations in our approach. In summary, our approach also uses 3D skeleton together with Markov model, but there are some differences compared with the two mentioned works. First, our work focuses on detecting abnormal gait in walking while studies [9,10] tried to build models of normal gait in different activities (e.g., walking up stairs, standing up and sitting down), thus our features for representing gaits are extracted based on only lower body joints instead of all possible joints. Second, HMMs with discrete observations are employed in our work instead of continuous ones for easier implementation and interpretation. In [11], skeleton poses were employed to detect abnormal gaits. The key of this research is that a set of consecutive skeletons is represented by a spatio-temporal feature, which is determined based on 3D position of joints together with the motion’s age. Although this approach provided good experimental results, it is difficult to obtain a similar accuracy in practical situations. Extracting each spatio-temporal feature based on a specific number of consecutive skeletons may lead to incorrect detection in subjects which perform the same gait but with different movement velocities.

Our approach aims to overcome the previously mentioned disadvantages. We assume that a human posture can be well assessed by the corresponding 3D skeleton, and a sequence of such skeletons can represent the gait and motion information. We then propose an approach to build a normal human gait model in order to detect anomaly in human walking. The input of our system is the human skeleton and 3D joints provided by the Microsoft Kinect. These parameters are determined based on the depth map estimated by emitting a structured infrared (IR) light pattern observed by a single IR sensitive camera (version 1 of the Kinect). The depth image is generated at 30 fps with acceptable resolution (640 by 480), and is not affected by changes of visible light. The posture corresponding to each frame is described by joint angles and related joint-planes of the lower body. The contributions of our work include (1) extracting relevant skeletal features that are helpful in distinguishing normal and abnormal gaits; (2) describing a simple gait cycle identification method; (3) using a simple and fast HMM-based algorithm with discrete observations, i.e., codewords, for online classification; (4) proposing a low-cost and easy to use Kinect-based gait analysis system for a clinical setting; and (5) confirming accuracy of abnormal gait detection by evaluating it on a large number of gait types from several databases and comparing it with a state-of-the-art study [10].

The rest of this paper is organized as follows: Section 2 explains the main idea for solving the abnormal gait detection problem; details of our approach are presented in Section 3; Section 4 shows experimental results and discussion; and conclusions are given in Section 5.

## 2. Sequential Abnormal Gait Detection

In a sequence of walking images, the most easily observed information is the instant gait at a specific time *t*, which is called atomic posture $p_{t}$. Typically, each such posture describes the orientation, position and relationship between pairs of consecutive bones of the walking person in the corresponding frame. It is the basic unit for representing human gait. However, extracting features related to all bones in the human skeleton would be unnecessary. From inspecting exemplary sequences of gait, it appears that relations between lower-body parts, including ilium, femur, shin, and legs, provide reliable cues to distinguish regular gait from any kind of anomaly. In most of normal gaits, the instant human posture satisfies some rules, e.g., joint angles fall in specific ranges depending on the type of joint of interest. Research and observations regarding human action analysis have shown that each person performs a walking pattern in a fairly repeatable way [12], e.g., the variation of each joint angle is relatively cyclical. In addition, periodic and symmetric movement of leg’s parts can be seen in normal walking motion, and the left and right limbs alternate their movement, whereas abnormal gaits tend to be more aperiodic and random.

Moreover, a walking anomaly may consist of a sequence of normal poses. In other words, the information of instantaneous posture taken alone at each frame may not be sufficient to determine the type (normal or abnormal) of gait because gait is a temporal phenomenon. For example, a person with a left leg pain could move this leg more slowly with respect to the right leg but each individual posture could still be normal. Therefore, we consider concatenated feature vectors corresponding to a gait cycle to incorporate the temporal context into the gait evaluation. Each such cycle is represented by a series of codewords that represent skeleton-based feature vectors extracted from $p_{t}$. A gait cycle is assumed as anomaly if the corresponding likelihood, which is estimated from an HMM model, is less than a learned threshold. Our proposed algorithm is presented in the next section.

## 3. Details of Our Approach

**Figure 1:**
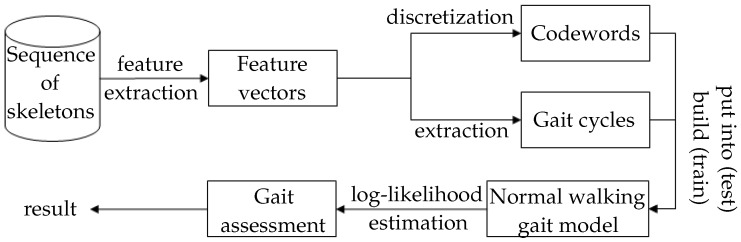
Flowchart of proposed methodology.

In this section, three main stages of our methodology are presented, including feature extraction, modelling, and gait assessment. An overview of this approach is shown in Figure 1.

### 3.1. Feature Extraction

**Figure 2:**
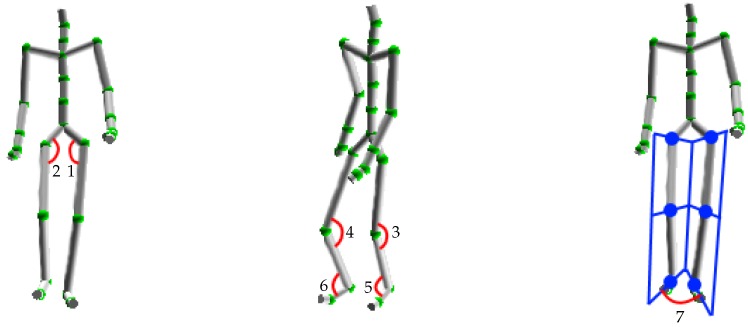
Positions of joints of interest and corresponding feature IDs.

In this stage, some properties are extracted in order to limit the feature space in representing a human posture. This posture is represented by a skeleton determined with the Kinect depth sensor [13]. Based on the provided 3D joints of the skeleton, we represent the human posture with the seven following features (see Table 1 and Figure 2):

The angles are employed to describe human pose in order to have invariant features with respect to the subject’s height and size. Because walking is essentially characterized by the lower-body motion, we consider only skeletal joints up to the waist. The angles of the first six features are estimated based on the 3D coordinates of three related joints provided by the Kinect. The seventh characteristic is a bit different. Two planes are first computed with the three joints of each leg. Then, the angle of interest is determined as the angle between the two normal vectors of these two planes. These seven values are elements of the feature vector that describes the human posture. In order to simplify gait representation, these vectors are discretized into a set of codewords.

Some related studies have employed upper body joints (e.g., [9,10]). However, we decided to focus only on lower joints for the following reasons. First, gait analysis based on lower-limb motion measurements is already well established in the clinical setting. We therefore expect that if there are any problems with a walking pattern (e.g., due to unbalance), the lower body joints will also be affected. Second, upper extremities show a very large working range with large variations in the normal population as opposed to the more typical lower limb gait pattern. In addition to increasing the complexity of the analysis, this may cause measurement problems in particular with the Kinect used in this study due, for instance, to self-occlusion (see Figure 5b,c where upper body joints were wrongly localized due to self-occlusion).

### 3.2. Vector Discretization

This stage is performed in order to represent similar postures with only one codeword. It allows some tolerance for small variations of a specific pose. The transformation from vectors into a collection of codewords is performed using a popular clustering technique, *k*-means [14], in which the number of clusters is chosen empirically. This algorithm groups feature points according to spatial (Euclidian) distance and is employed because of its simplicity and efficiency. Each cluster is assigned a number, and vectors that fall within this cluster are converted into the codeword with the same value.

### 3.3. Gait Cycle Extraction

The extraction process is performed with the support of a parameter that has the same periodicity as the gait cycle. The spatial distance between the two feet is employed in our approach with a smoothing filter to remove noise. Exponential smoothing [15] is employed with the general formulas:

(1) $$s_{t}=\left\{\begin{array}{cc} x_{t}, & t=0,\\ \alpha x_{t}+\left(1-\alpha \right)s_{t-1}, & t>0, \end{array}\right.$$

where *α* is the smoothing factor (0<α<1), *t* is the frame index, $x_{t}$ and $s_{t}$ are the raw and smoothed distance values, respectively.

**Figure 3:**
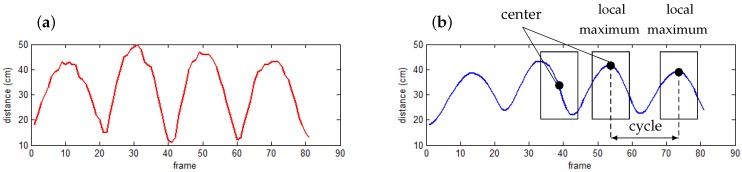
Cycle determination from a sequence of distances between two feet: (**a**) raw distance values; and (**b**) smoothed distance values (α=0.2).

After getting the waveform values, each gait cycle is estimated by a pair of two consecutive local maxima, which are determined using a sliding window of fixed length. This algorithm is attractive because it is not only simple and intuitive, but also an online algorithm. At time *t*, the sliding window considers a sequence of smoothed distances, which consists of values from time (t−n+1) to *t*, in which *n* is the window length. If the center value is the maximum of this sequence, the corresponding human posture is assumed as a starting/ending point of a cycle. In some cases, a local maximum is found because of noise during data acquisition. This limitation could be avoided using statistical parameters such as standard deviation together with a threshold, or regression techniques [16]. An illustration of gait cycle detection is shown in Figure 3.

### 3.4. Normal Gait Modeling

There are various techniques for solving a recognition problem that could be categorized as template matching or model-based methods. The former solution is not employed in our approach because sequences of codewords, corresponding to gait cycles, have various lengths. A matching technique, e.g., dynamic time warping (DTW) [17], could deal with this property by stretching and/or compressing cycles in order to perform the comparison; however, this results in a loss of information since a long gait cycle can be an indication of anomaly. Therefore, a model-based methodology was chosen in our work.

**Figure 4:**
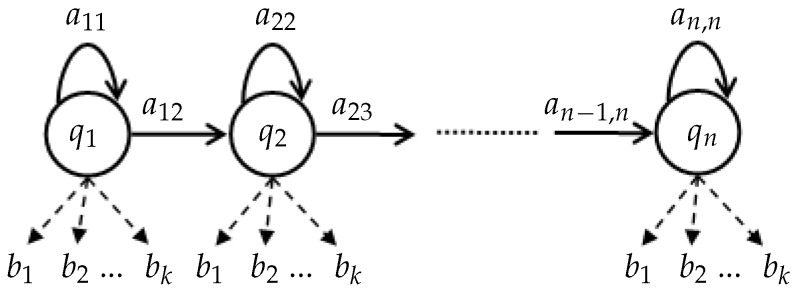
HMM (hidden Markov model) architecture with *n* states $q_{i}$, $a_{ij}$ is the state transition probability from state $q_{i}$ to $q_{j}$. Each *b* value corresponds to a label representing a human posture that is obtained in the vector discretization stage.

Our model is built using a training set of cycles corresponding to normal gait. The modeling process is implemented using hidden Markov model (HMM) [18] because this technique is effective in describing the transition of human posture states during a gait cycle. Another reason is that our model must deal with gait cycles, which may have different lengths (e.g., walking with various velocities) represented as vectors of various length, well-known techniques such as support vector machine or neural network, and, thus, are not appropriate. Our HMM architecture is illustrated in Figure 4, in which the number of observations (codewords) is determined in the vector discretization stage.

Notice that there is no transition from $q_{n}$ to $q_{1}$ in our work because of the following reasons. First, we process each gait cycle separately using a gait cycle extraction step (Section 3.3); therefore, a sequential HMM is more appropriate in that case. Second, if a transition from $q_{n}$ to $q_{1}$ was assigned, the sequence of states would become more unpredictable since the HMM would be circular and any state could be the initial state and the final state which could decrease the performance. Third, all postures could occur in all states with different probabilities, thus it was not necessary to give a direct relation between $q_{1}$ and $q_{n}$. In summary, we tried to represent each gait cycle in a specific order of postures in a vector, and thus the first and last states were separated, although they might represent the same posture.

### 3.5. Threshold Estimation

This step is performed after obtaining the model. The normality threshold is estimated based on the mean and standard deviation of log-likelihood of training samples as follows:

(2) $$threshold=\mu +\lambda \sqrt{\frac{\sum_{i=1}^{n}{\left(\zeta_{i}-\mu \right)}^{2}}{n}},$$

where *λ* is a constant, *n* is the number of training patterns, and *ζ* and *μ* are the log-likelihood and mean of such values, respectively. Note that *μ* and *λ* are negative values due to the fact that log-likelihoods $\zeta_{i}$ are less than zero. A gait cycle is considered as an anomaly if the corresponding log-likelihood is less than the threshold. In practical systems, more robustness could be implemented by requiring a minimum number of abnormal gait cycles through a specified period of time to confirm that the anomaly has been occurred.

## 4. Experiment

**Figure 5:**
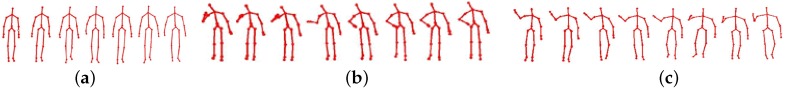
Three gait types captured by the Kinect: (**a**) normal gait; (**b**) left–right asymmetry gait; and (**c**) hunched back gait. Notice that the arms were sometimes localized incorrectly by the Kinect, but it did not affect our processing since only the lower-body parts were analysed.

Our testing system was implemented in C# language using an Accord.NET Framework [19]. We employed two datasets including a collection of mocap data and sequences of Kinect skeleton. The former is provided in [20], while the latter was recorded in our laboratory by five volunteers (Subjects 1–5 in Table 2) with different body shapes in a realistic environment. The mocap represents eight gait types including normal and seven abnormal ones obtained from 20 subjects. The number of walking cycles in this dataset is 2137 according to our method in Section 3.3. Our Kinect dataset, which was captured on a straight walkway, consists of three gait types which are normal, left-right asymmetry, and hunched back gaits. The first gait type was recorded when volunteers were walking normally. The second one was acquired with a simulated pain in the knee. In this case, the injured knee could not flex when walking normally. Therefore, the body centroid tended to tilt on the other leg, and the stride length was significantly different between the two legs. The third gait type was performed with a simulated low back pain, in which the human body bended forward and one hand was placed behind the back, and thus the body centroid was more forward and switched continuously between the two legs during walking. Note that the arms were sometimes localized incorrectly by the Kinect, but it did not affect our processing since only the lower-body parts were analysed. These three gait types are illustrated in Figure 5.

The number of gait cycles corresponding to each gait type is presented in Table 2. Note that these cycles were decomposed using our proposed technique, unavailable in the two datasets. It is obvious that mocap systems certainly provide less noisy data than the Kinect. However, these gait measurement systems are costly and generally require manual examination, calibration procedures, and the precise placement of sensors/markers on the body of the patient. On the contrary, depth cameras such as the Kinect provide a low-cost, markerless and calibrationless system alternative that is very promising for gait analysis as well as more general 3D human motion assessment. Consequently, our system is low-cost, and easy to use and offers a promising tool for a wide range of applications for gait analysis. As will be demonstrated in this section, the proposed approach can cope with the noisier data generated by the Kinect. In addition, other studies such as [9,10] have worked successfully with depth camera for gait analysis.

**Figure 6:**
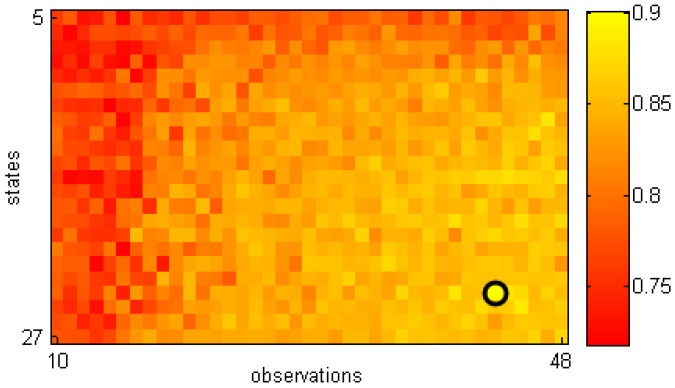
Gait assessment accuracies when testing different HMMs.

The model of normal walking gait was built based on 3/4 the number of normal gait cycles recorded by Kinect, and were selected randomly. Our testing process was performed on the remaining normal gait cycles and all abnormal gait types (445 normal cycles and 2208 abnormal cycles). In our experiment, the smoothing factor *α* in Equation (1) was set to 0.2 in order to remove noise while keeping the global shape of the graph of distance values. The constant *λ* in Equation (2) was set to −1.28, which corresponds to the 10th percentile for a Gaussian distribution. The size of the sliding window in our system was assigned to 5. The optimized solution for HMM parameters was found empirically by testing our approach with different sets of HMM observations (equal to *k* in the clustering step) and states in order to obtain the best classification accuracy. The overall results corresponding to 897 HMMs are shown in Figure 6. The best accuracy was 90.12% when the model had 43 observations (codewords) and 24 states. Using a larger number of states and/or observations would also increase the computational cost, thus impairing online detection. In these experiments, our system gave an online classification result immediately after completion of the gait sequence. In addition, our HMMs still work well with new subjects, i.e., without gait template in the training set.

**Figure 7:**
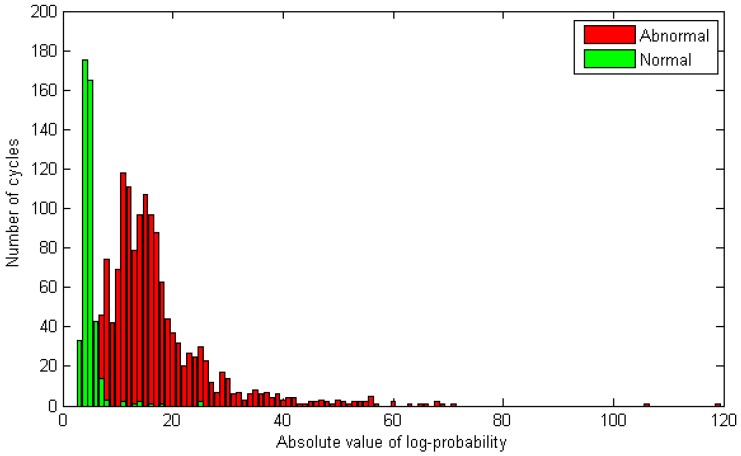
Absolute values of log-probabilities, |ζ| in Equation (2), calculated from HMM output. **Green** and **red** color represent normal and abnormal gaits, respectively. The threshold is estimated in the training process. Some samples are not shown in this figure because they were outside the range displayed here.

From now on, we consider only the best model. The absolute values (for better visual illustration) of log-probabilities corresponding to normal and abnormal gaits are shown in Figure 7.

**Figure 8:**
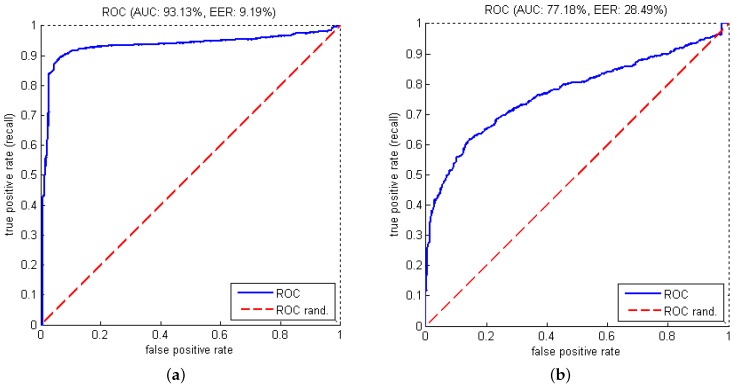
ROC (Receiver Operating Characteristic) curves of abnormal gait recognition corresponding to the mentioned best HMM and the DTW (Dynamic Time Warping) with same number of observations. (**a**) abnormal gait detection using HMM; and (**b**) abnormal gait detection using DTW.

The correct detection of normal cycles (specificity) and abnormal data (sensitivity or recall) were 93.48% and 89.44%, respectively. Precision, F1-score, EER (Equal Error Rate) and AUC (Area under the ROC (Receiver Operating Characteristic) curve) are also shown in Table 3. The ROC curve generated with different thresholds is displayed in Figure 8a.

As shown in Table 3, the ability of gait assessment with the Kinect data was slightly less than the mocap one, but was still quite good. In other words, these measures confirm that our approach can cope with the noisy data generated by a low-cost device such as the Kinect.

Some studies on problems related to temporal factor such as gesture classification [21] and word recognition [22] showed that DTW outperforms HMM in their experiments. Therefore, in our work, we also performed a test with DTW in order to provide a comparison with our HMM-based method. In this test, the clustering stage was done with 43 clusters (similar to the best HMM in our experiments). The obtained results are represented as the ROC curve in Figure 8b. It is obvious that our HMM is significantly better than DTW in detecting abnormal human gaits. A detailed study for comparing HMM and DTW on different problems would be interesting in the future.

We also performed some experiments on the dataset SPHERE-Walking2015 used in [10] that includes 40 sequences of 10 individuals with normal and abnormal (stroke and Parkinson) gaits on a flat surface. However, the skeleton of that dataset contains only the 15 joints provided by OpenNI SDK (Software Development Kit) (we had 20 joints with the Kinect version 1 SDK). This means that some of our features (#5 and #6) could not be measured. Nevertheless, we conducted experiments with the five remaining features and have reported our results in Table 4.

**Figure 9:**
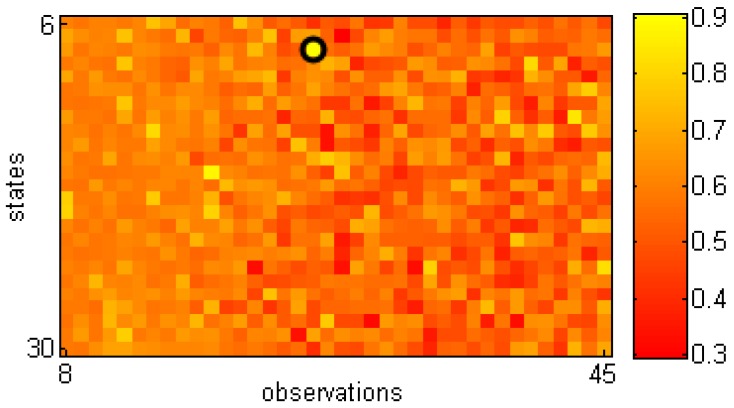
AUCs (Area Under Curve) corresponding to gait assessment results when testing with different HMMs. Vertical and horizontal axes are numbers of states (in range 6–30) and observations (in range 8–45), respectively. Model with the highest value is highlighted.

We used the same training and testing sets as in the work [10], but cycle segmentation was performed based on our approach since the dataset does not provide them. The method in [10] evaluates the whole sequence while our approach process each gait cycle separately. This means that we had to extract each gait cycle first (Section 3.3). It also means that for any sequence, we got as many evaluations as there were gait cycles. Therefore, to compare with the results in [10], in our experiments, mean values of log-likelihoods corresponding to three consecutive cycles in each sequence were computed, and the smallest one was used to represent each sequence. This is a reasonable choice since it looks for a significant abnormality within the sequence for providing a decision. Furthermore, this can be applied on sequences of various lengths in term of the number of gait cycles. The value of 3 was set to reduce the effect of noisy cycles. As in [10], AUCs with different HMMs (in terms of the number of states and observations) were computed and are shown in Figure 9. The highest AUC for assessing the gait types was 0.91 with eight states and 25 observations.

Some statistical measurements corresponding to the best accuracy obtained from the mentioned HMM are given in Table 4. These overall results show that our approach could provide good gait assessment. Table 5 shows AUCs reported in the work [10] on the same dataset for the best models. This measure ranged from 0.79 to 1.00 with different HMM types, manifold dimensions, and feature ensembles. However, notice that the AUCs were not computed in the same way for both methods. The measure of [10] is harsher and therefore their results would appear artificially lower. Because our normal gait models for this dataset were built based on five of the seven proposed skeletal features, our approach thus could be expected to retrieve better results when working with the full lower body skeleton. Another comparable aspect between our study and the work [10] is the speed of execution. Since all models and experiments in [10] were done using Matlab (2012b, MathWorks, Cambridge, MA, USA), we rebuilt our system in this language and computed the average processing time per frame. The obtained value on a laptop with an Intel i5-6200U CPU 2.8 GHz processor (Santa Clara, CA, USA) and 12 GB RAM was 0.4 ms, while the corresponding measures in [10] with a more powerful workstation (Intel I7-3770S CPU 3.1GHz processor and 8 GB RAM) were 15.99, 16.27, 30.16 and 153 ms with increasing model complexity. The main reasons for our faster computation are that (1) extracted features were significantly simpler with less joints compared to [10]; (2) discrete observations were used in our work instead of continuous one; and (3) our model estimated the likelihood on each gait cycle independently without using all other previous frames. Therefore, our approach could be suitable for practical applications that require a fast computation.

## 5. Conclusions

In this paper, we propose an approach for abnormal gait detection without prior knowledge about anomaly in human walking. Our work focuses on a low-cost and easy to use gait analysis system for a clinical setting. This system is fully automated, with no markers or sensors on the patient’s body, no calibration and no manual intervention. It can detect abnormal gaits and provide a normality index (likelihood) if needed. In addition to neurological/musculoskeletal disorder screening, it could enable clinicians to perform a follow-up of patient’s recovery after surgery, treatment (e.g., joint replacement) or after a stroke. Our results show that our system is a promising tool for gait analysis. This method is applicable to many different gait types. The contributions of our work include (1) extracting relevant skeletal features which are helpful in distinguishing normal and abnormal gaits; (2) describing a simple gait cycle identification method; (3) using a simple and fast HMM-based algorithm with discrete observations, i.e., codewords, for online classification; (4) proposing a low-cost and easy to use Kinect-based gait analysis system for a clinical setting; and (5) confirming accuracy of abnormal gait detection by evaluating it on a large number of gait types from several databases and comparing it with a state-of-the-art study [10]. Our system consists of four stages. First, seven joint-related features are extracted from the human skeleton in each frame. Second, the *k*-means clustering technique is employed to perform vector discretization. The cycle extraction step is then implemented based on the distance between the feet. Finally, a model of normal gait and a threshold is created in order to assess the normality level (likelihood) of a gait cycle. Experiments on 10 types of human gait, including normal and nine different abnormal ones as well as on datasets in the work [10] show that our model provides excellent results for distinguishing normal and abnormal walking gaits in terms of performance metrics (see Table 3 and Table 4 with a dataset of [10]). For instance, usually a AUC greater than 0.9 is considered as excellent classification. This is comparable to the best state-of-the-art algorithms such as [10]. Besides the ability of gait assessment, the fast computation speed is also an advantage compared with [10]. In addition, our method provides immediate results to inform the patient after his/her gait test to help relieve anxiety and for those who are diagnosed as abnormal to quickly start treatment or further analysis. It also allows screening more patients at a minimum cost. Finally, our method could be easily integrated within a larger gait analysis system since it is not time-consuming.

In summary, the experiment shows that our system provides an excellent efficiency in identifying anomalies in human walking. However some modifications could be done to improve the recognition accuracy. For instance, in some testing cases of the mocap dataset, a normal gait was detected as an anomaly when the corresponding walk trajectory was significantly curved (e.g., a performer changes his/her walking direction at a room corner). Such curved-trajectory cycles could have been ignored, since, in a clinical application, the patient would typically be required to walk in a linear walkway. In addition, some short-length cycles were wrongly detected as abnormal because of the effect of noise in localizing foot joints. A threshold value could be defined in order to eliminate or incorporate each such cycle into the next one. More robustness could be added to the proposed method by requiring a minimum number of abnormalities through a specified period of time to confirm the anomaly. Finally, our algorithm does not require the normal gait pattern of each subject since it *knows* the gait pattern of a normal population. Notice that the HMM could be trained with a larger normal dataset to further improve its performance in the future.

As future work, some other skeleton-based characteristics will be assessed (e.g., upper body). Principal component analysis, linear discriminant analysis or other machine learning algorithms could be employed in order to select better features. Our proposed methodology will also be improved to create specific models of different pathological gaits, such as choreiform, hemiplegic, diplegic, and Parkinsonian [23]. In addition, other classification techniques, e.g., regression [24], will be investigated for comparison to choose the most appropriate model for each gait-related problem.

## Figures and Tables

**Table 1:** Features of interest which describe the human posture.

ID	Feature
1	Left hip angle
2	Right hip angle
3	Left knee angle
4	Right knee angle
5	Left ankle angle
6	Right ankle angle
7	Two feet angle

**Table 2:** Components of two datasets used in experiments.

Subject	Normal	Hunched Back	Left-Right Asymmetry	Headache	Arthritic	Walk One Leg	Shuffle One Leg	Stealth	Stomachache	Zombie
01	26	42	41	-	-	-	-	-	-	-
02	43	48	48	-	-	-	-	-	-	-
03	68	59	56	-	-	-	-	-	-	-
04	27	45	46	-	-	-	-	-	-	-
05	21	48	35	-	-	-	-	-	-	-
06	32	-	-	40	-	-	27	-	-	37
07	34	-	-	33	29	61	43	19	41	31
08	-	-	-	24	-	-	-	-	-	33
09	41	-	-	-	35	-	44	31	-	36
10	16	-	-	-	-	16	10	17	-	-
11	-	-	-	-	-	43	20	20	-	-
12	-	-	-	-	-	42	24	25	-	-
13	-	-	-	-	-	29	25	-	-	-
14	19	-	-	-	-	24	-	13	-	-
15	24	-	-	-	-	-	-	-	-	-
16	19	-	-	-	-	33	18	22	-	-
17	36	-	-	-	-	34	32	33	-	-
18	32	-	-	-	-	53	-	32	-	41
19	42	-	-	-	-	68	46	38	-	36
20	-	-	-	-	-	75	-	-	44	-
21	43	-	-	-	-	-	30	26	-	-
22	27	-	-	-	-	-	36	27	-	24
23	32	-	-	-	-	-	35	-	-	-
24	-	-	-	-	-	-	-	22	-	-
25	-	-	-	-	-	-	-	-	-	63

**Table 3:** Precision and recall on detecting abnormal gaits.

Dataset	Precision	Recall/Sensitivity	Specificity	F-Measure	Accuracy	AUC	EER
Kinect + Mocap	0.986	0.894	0.935	0.938	0.901	0.931	0.092
Kinect only	0.990	0.853	0.917	0.916	0.859	0.887	0.098
Mocap only	0.984	0.906	0.937	0.943	0.912	0.938	0.089

**Table 4:** Best result when testing on the dataset SPHERE-Walking 2015 (University of Bristol, Bristol, United Kingdom) [10].

Precision	Recall/Sensitivity	Specificity	F-Measure	Accuracy	AUC	EER
0.938	0.882	0.800	0.909	0.864	0.91	0.20

**Table 5:** AUC (Area Under Curve) results reported in the work [10] on the dataset SPHERE-Walking 2015 with different skeletal features, dimension reductions, and measures for the best models $\lambda_{c}\left(\vartheta_{t}\right)$ and $\lambda_{d}\left(\vartheta_{t}/\vartheta_{\omega t}\right)$, in which $\lambda_{c}$ and $\lambda_{d}$ are HMMs with discrete and continuous states, $\vartheta_{t}$ and $\vartheta_{\omega t}$ correspond to measures on a frame and within a window, respectively (see [10] for details).

Motion Model	Feature	Manifold Dimension				
		1	2	3	4	5
$\lambda_{c}\left(\vartheta_{t}\right)$	JP	0.96	1.00	0.99	1.00	1.00
	JV	0.93	0.79	0.86	0.95	0.85
	PJA	0.91	0.98	1.00	0.96	0.98
	PJD	1.00	1.00	1.00	1.00	1.00
$\lambda_{d}\left(\vartheta_{t}/\vartheta_{\omega t}\right)$	JP	0.96/1.00	0.99/1.00	0.95/1.00	0.99/1.00	0.93/1.00
	JV	0.95/0.98	0.88/0.86	0.87/0.95	0.91/0.95	1.00/1.00
	PJA	0.89/0.91	0.82/0.88	0.91/0.96	0.94/0.96	0.94/0.96
	PJD	0.96/1.00	0.91/0.96	0.92/0.95	0.89/0.93	0.93/0.98

## Abbreviations

The following abbreviations are used in this manuscript:

HMM	Hidden Markov Model
DTW	Dynamic Time Warping
RGB	Red, Green, Blue
ROC	Receiver Operating Characteristic
AUC	Area Under Curve
EER	Equal Error Rate
SDK	Software Development Kit
